# *Escherichia coli* as host for membrane protein structure determination: a global analysis

**DOI:** 10.1038/srep12097

**Published:** 2015-07-10

**Authors:** Georges Hattab, Dror E. Warschawski, Karine Moncoq, Bruno Miroux

**Affiliations:** 1 Laboratoire de Biologie Physico-Chimique des Protéines Membranaires, Institut de Biologie Physico-Chimique, CNRS, Univ Paris Diderot, Sorbonne Paris Cité, PSL research university, Paris, France

## Abstract

The structural biology of membrane proteins (MP) is hampered by the difficulty in producing and purifying them. A comprehensive analysis of protein databases revealed that 213 unique membrane protein structures have been obtained after production of the target protein in *E. coli*. The primary expression system used was the one based on the T7 RNA polymerase, followed by the arabinose and T5 promoter based expression systems. The C41λ(DE3) and C43λ(DE3) bacterial mutant hosts have contributed to 28% of non *E. coli* membrane protein structures. A large scale analysis of expression protocols demonstrated a preference for a combination of bacterial host-vector together with a bimodal distribution of induction temperature and of inducer concentration. Altogether our analysis provides a set of rules for the optimal use of bacterial expression systems in membrane protein production.

Membrane proteins (MP) play a central role in several biological processes, which includes cell signaling, ion and metabolites transport, and energy conversion. Since the first MP structure was determined in 1986^1^, over 450 unique MP structures have been obtained (see crystal structure list from White^2^, and NMR structure list from Warschawski^3^). They provide molecular details explaining how MP work. Despite numerous breakthroughs in X-ray diffraction^4^,^5^, NMR^6^ and electron microscopy^7^, as well as in MP production^8^,^9^,^10^,^11^ and stabilization^12^, the structural biology of MP is hampered by the production of the recombinant protein and its purification in a functional state. In 1986, Studier and colleagues^13^ set up a powerful bacterial expression system, in which the RNA polymerase from the bacteriophage T7 specifically drove the transcription of the target gene inserted in the expression plasmid, under the control of the T7 promoter. However, one of the main drawbacks of the T7 based expression system is that the rate of transcription of the target gene is rather fast because the T7 RNA polymerase (T7 RNApol) transcription activity is over ten times faster than *E. coli* RNA polymerase. Moreover, the expression system is further enhanced by the copy number of the expression plasmid. Consequently, upon expression of the T7 RNApol, an excess of target RNA is produced, which is often toxic to the cell, and triggers uncoupling between transcription and translation and growth arrest^14^,^15^. Therefore, several strategies have been developed to attenuate and better regulate the T7 expression system: 1. Introducing a T7/*lac* hybrid promoter within the expression plasmid; 2. Over-expressing the T7 lysozyme, a natural inhibitor of the T7 RNApol^16^; 3. Expressing the T7 RNApol under the control of the tightly regulated arabinose promoter (BL21-AI, Invitrogen).

In 1996, two spontaneous mutants of the BL21λ(DE3) bacterial host, namely C41λ(DE3) and C43λ(DE3), were isolated by exploiting the toxicity of the over-expression of the oxoglutarate mitochondrial carrier gene and of *atpF* encoding the b subunit of the *E. coli* ATP synthase, respectively^17^. We found that the level of accumulation of the target gene mRNA is ten times lower in C41λ(DE3) and is delayed by one hour in the C41λ(DE3)-derived bacterial host, C43λ(DE3). More recently, Wagner *et al.* have shown that the level of T7 RNApol is strongly reduced in both mutants, thereby allowing the bacterial cell to mediate cell growth with protein production^18^. Ensuring viability allowed metabolic adaptation, as illustrated by the production of the b subunit of the ATP synthase. In the C41λ(DE3) host, the b subunit was found in a partially unfolded state whereas in the C43λ(DE3) host, the production of the protein was accompanied by intense membrane proliferation with the b subunit in the correctly folded state^19^. In parallel to developments in the T7 based expression system, alternative expression systems have been established by employing arabinose^20^, lactose, tetracycline^21^, or T5 promoters^22^. Today, a profusion of expression plasmids and bacterial hosts are available for protein over-production^23^,^24^, however there is no clear rationale for choosing the appropriate bacterial expression system in each individual case.

Our objective here was to perform a global analysis of existing expression systems in the frame of MP production and structure determination. In a first step, entry codes referring to membrane protein structures obtained from *E. coli* were extracted from the Protein Data Bank (PDB) and the two major expression systems, T7 and arabinose promoter based, were identified. In a second step, a bibliographic database was constructed to perform an extensive analysis of expression protocols. The results we have obtained thus provide a systematic set of rules for the successful production of membrane proteins in *E. coli*.

## Results

### Analysis of bacterial expression systems for MP structure determination

At the time of the analysis, June 2014, 213 unique MP structures (including 72 *Escherichia coli* MP, see supplementary Tables 1 and 2) were retrieved from the crystallographic^2^ and NMR^3^ databases on the basis of having been produced in *E. coli.* First, we focused our analysis on the heterologous production of MP in *E. coli*. Table 1 summarizes the distribution of the 163 expression vector/bacterial hosts used for obtaining the 141 unique non *E. coli* MP structures. The first remarkable observation is that the T7 promoter based expression system is dominant (63%) followed by the arabinose, *tac* and T5 promoter based expression systems (17%, 9% and 7%, respectively). The pASK tetracycline induced expression vector also shows a detectable impact (5%), which is notable given that it is exclusively marketed by a small biotech company (IBA, Goettingen Germany). Within the T7 based expression system, five bacterial hosts, namely BL21λ(DE3), C41λ(DE3), C43λ(DE3), BL21λ(DE3)-pLysS, and BL21λ(DE3)-CodonPlus are extensively used (91%). The bacterial host BL21(DE3) is first (40 MP structures), followed by the two mutant hosts, C41λ(DE3) and C43λ(DE3) (16 and 18 MP structures respectively), and then the combination of BL21λ(DE3) with a companion plasmid expressing either lysozyme or a rare tRNA (12 and 7 MP structures respectively). Bacterial hosts other than those mentioned above have only had a marginal impact in the field (1 to 2 MP structures).

**Figure 1:**
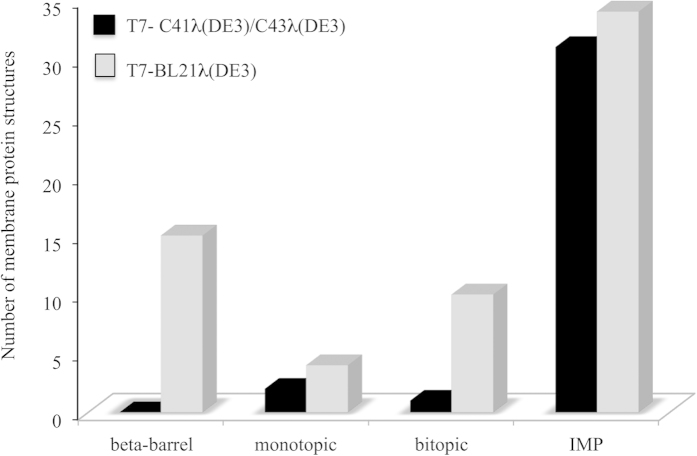
Distribution of secondary structures in MP structures within the T7 expression system. Membrane protein structures obtained from overexpression in the T7 system (102 see Table 1) were classified according to their secondary structure and topologies. For α-helical membrane proteins the number of transmembrane spans was represented as follow: monotopic (without transmembrane span), bitopic (1 transmembrane span) and integral membrane proteins (IMP, more than 1 transmembrane α-helices).

Next we asked whether some bacterial hosts had more success with integral membrane proteins (IMP), which are the most difficult class of membrane proteins to express. Non *E. coli* MP structures obtained within the T7 expression system were classified according to their secondary structures and topologies. As shown in Fig. 1, half the α-helical integral membrane protein structures obtained so far are produced either in C41λ(DE3) or C43λ(DE3) (14 and 17 IMP, respectively). Within this IMP group, distribution of the number of MP transmembrane spans is independent of the bacterial host (supplementary Figure 1). In contrast, no β-barrel MP are produced in the T7 based expression system with those mutant hosts (Fig. 1). In the arabinose promoter based expression system (Table 1), the bacterial host C43λ(DE3) surprisingly appears as the best choice (10 MP structures, including 7 β-barrel MP), followed by the *BL21*-*T1*^R^ and TOP10/DHB10 hosts (4 and 3 MP structures respectively). The bacterial strain XL1-Blue is preferred when using the T5 promoter system (4 MP structures). Next, we determined whether the success of the C41λ(DE3) and C43λ(DE3) mutants is specific to heterologous production of MP, or if it also impacted the homologous production of MP.

Table 2 shows the distribution of expression hosts and promoter, for 72 unique MP structures found in the PDB. The greater number of unique expression systems, 111, for producing only 72 unique MP structures, suggests that, in contrast to the heterologous production of MP, the choice of the promoter and the expression host is more flexible. For instance, the crystal structure of OmpG was obtained after production of the protein in C43λ(DE3) with an arabinose promoter expression plasmid (2F1C^25^), or in C41 λ(DE3) (2IWW^26^) and in BL21λ(DE3)pLysE (2JQY^27^) with a T7 based expression plasmid. Similarly, the lactose permease was expressed under the control of its native promoter in the XL1Blue host grown in a 150l fermenter (1PV7, 2CFQ^28^,^29^), and more recently using either C41λ(DE3) or C43λ(DE3) with a T7 based expression plasmid^30^,^31^ (see supplementary Table 2). Table 2 also shows the impact of the T7 system (60%) and of native promoters (18%). For example, OmpC (2J1N^32^), LamB (1MPM^33^), AcrB (2RDD^34^), and the electron-transfer chain complex 1 (3M9C^35^), were produced under the control of their own promoter in multi-copy plasmids (Supplementary table 2). Within the T7 based expression system, the leading host is BL21λ(DE3) (28 MP structures) followed by C43λ(DE3), C41λ(DE3) and BL21λ(DE3)pLysS (10, 7, and 6 MP structures, respectively).

Overall, both C43λ(DE3) and C41λ(DE3) mutant hosts contributed to 28% of non *E. coli* MP structures and 19% of *E. coli* MP structures deposited into the PDB.

### Analysis of T7 and arabinose based expression protocols

**Figure 2:**
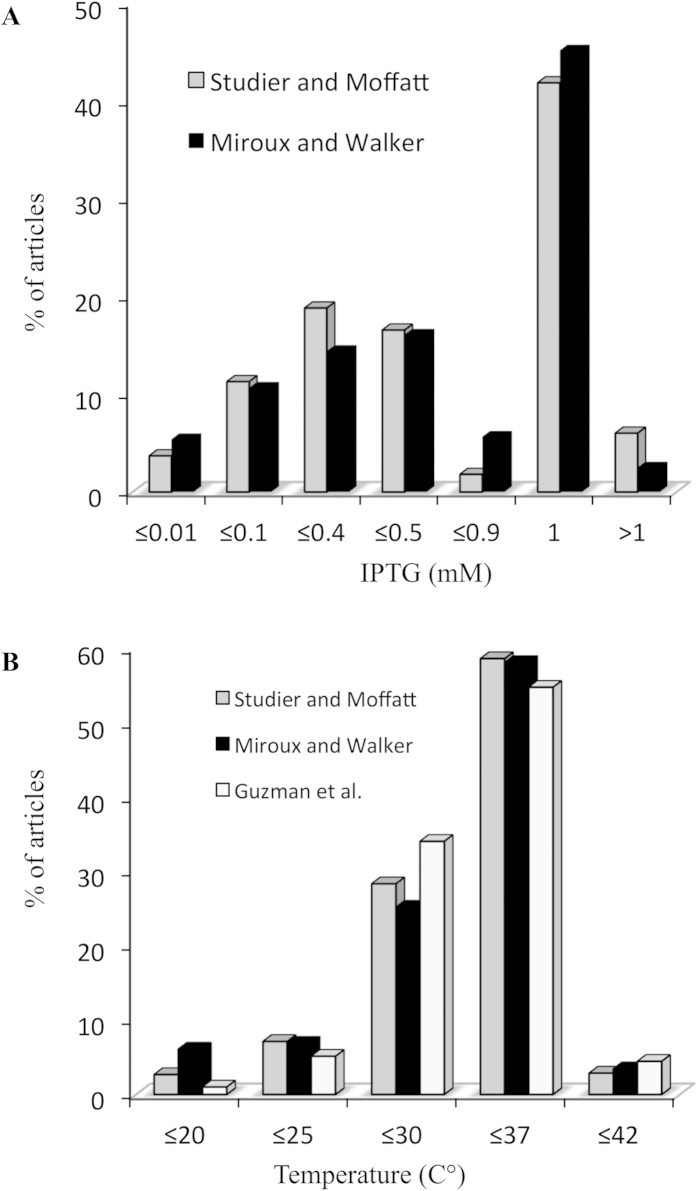
Expression protocol parameters in T7 and arabinose based expression systems. Inducer concentrations and temperatures of growth were extracted using regular expression patterns in articles citing either Miroux and Walker, Studier and Moffatt or Guzman *et al.* for the recombinant expression of proteins in *E. coli* (see Materials and Methods). **A.** ITPG concentration in the T7 based expression system; **B.** temperature of growth in both T7 and arabinose based expression systems. Data are expressed as percentages of the total number of articles where an explicit value was found (See Table 2).

A database was constructed, containing 2817 articles citing either Miroux and Walker^17^, Studier and Moffatt^13^, or Guzman *et al.*^20^, for the use of C41λ(DE3)/C43λ(DE3) (group 1), Bl21λ(DE3) (group 2) bacterial hosts in the T7 based expression system, or any bacterial host in the arabinose inducible promoter system (group 3), respectively. All groups were first parsed with the regular expression “membrane protein”, which was found in 77% of the articles in group 1, 25% of articles in group 2 and 45% of articles in group 3 (Table 3). Next, we investigated expression protocols, focusing on inducer concentration and growth temperature (see Materials and Methods section). Explicit values for IPTG concentration or growth temperature were unstated in half of the articles (Table 3). Fig. 2 shows a bimodal distribution for both parameters. As expected, the majority of articles refer to 1mM IPTG concentration as the induction condition and 37 °C growth temperature. However, in 50% of the cases, IPTG concentration is below 0.5 mM, and in 38% of the cases growth temperature is equal to, or below, 30 °C (Fig. 2). Of note, a significant number of publications, 94 altogether from groups 1 and 2, refer to IPTG concentration below 10 μM, which is consistent with the recently published improved induction protocol at 8 μM IPTG^36^. Accordingly, we found two MP structures where the recombinant protein was obtained without any addition of IPTG in BL21λ(DE3) cell cultures (2PRM^37^, 4EIT^38^).

Next, the distribution of plasmids in all three groups of articles was investigated (Table 3). Frequency citation of T7 based high copy number plasmids is greater in group 1 (19% versus 13% in group 2), whereas attenuation plasmids are less cited (10% versus 16% in group 2). Arabinose based promoter pBAD plasmids increased in group 1 (9% versus 3% in group 2), which correlates well with the significant number of MP structures obtained with the C43λ(DE3)/pBAD host-expression vector system combination (10 and 4 MP structures in Tables 1 and 2 respectively). Citation of pBAD plasmids were found in 99% of group 3 articles. The use of pRARE companion plasmids supplementing rare tRNA is marginal in all three groups, which is consistent with the small number of MP structures that are produced with the BL21λ(DE3) CodonPlus bacterial host (7, see Table 1). To confirm the link between high copy number plasmids and C41λ(DE3) and C43λ(DE3) bacterial hosts, a subset of the bibliographic database comprising frequent users of those mutant hosts, which was identified by the number of citations in group 1, was further analysed. Table 4 summarizes plasmid usage from eight laboratories representing 124 citations. In this subset, the use of the C43λ(DE3) bacterial host was low (13%) and exclusively associated with low copy-number vectors. In more than half the studies (53%), high copy number plasmids (pRSET from Invitrogen or pHis/pMW7 expression vectors 39,40) were used in combination with C41λ(DE3).

## Discussion

One of the primary objectives of this study was to assess the impact of bacterial expression hosts for membrane protein structure determination. We found that 28% of all non *E. coli* MP structures have been resolved from MP produced from the C41λ(DE3) and C43λ(DE3) bacterial hosts, which has been distributed since 2000 by Lucigen. Thus these hosts, together with the parental host BL21λ(DE3), have significantly contributed to the success of bacterial expression systems in structural biology. In contrast, other expression systems have had moderate or little impact on the field, most likely because they may have failed to provide sufficient amount of protein for structural studies. Significantly, both mutant hosts have been used for MP difficult to produce in large amounts. For instance, they have been used mainly to produce α-helical MP, which comprise 50% of non *E. coli* MP within the T7 based expression system, rather than being used to produce beta-barrel proteins, which can be produced by almost any expression system (see OmpG, supplementary Table 2). Regarding bitopic MP, essentially produced in the BL21λ(DE3) bacterial host, it should be noted that in all cases but one (3VMT^41^, a glycosyltransferase from *Staphylococcus aureus*), a small truncated form of the protein (usually 30–60 amino-acids), excluding the soluble domains, was produced in *E. coli* for NMR studies.

One explanation for the success of the C41λ(DE3) and C43λ(DE3) bacterial hosts is that there is improved regulation in the expression of the target gene. Before induction, the basal level of expression of the target gene mRNA is undetectable, but the strength of the expression system is not compromised after induction^17^. Secondly, the decrease in accumulation of the target mRNA in those mutants improves the coupling between transcription and translation^42^, a critical issue for high-level production of some MP^43^. Wagner *et al.* have demonstrated that in the C41λ(DE3) and C43λ(DE3) mutant hosts the level of T7 RNA polymerase is decreased tenfold^18^, which is a reasonable explanation for the improved regulation in this system. Similarly, co-production of the lysozyme has been shown to inhibit the activity of the T7 RNA polymerase^16^, which thereby reduces the basal and induced amount of enzyme available for the transcription of the target gene. Recently, the Cole group isolated new bacterial derivatives of BL21λ(DE3)^44^. The authors used the chemotaxis protein CheY fused to GFP as a model. The level of the CheY-GFP fusion protein was increased by 25% in their most potent BL21λ(DE3) derivative, P2-Bl21λ(DE3), as compared to the C41λ(DE3) host. At the chromosomal level however, the situation is still unclear. Wagner *et al.* have shown that in C41λ(DE3) and C43λ(DE3) the *lacUV5* promoter, which drives the expression of the T7 RNApol gene within the lambda DE3 insertion, has been replaced by the natural *lac* promoter, likely due to homologous recombination with the genomic copy of the *lac* operon^18^. Cole *et al.* found the same mutation in the T7 promoter but they postulated that additional mutations account for the production levels of the CheY-GFP fusion protein between P2-BL21λ(DE3) and C41λ(DE3). The secondary mutation in C43λ(DE3), which derives from C41λ(DE3), is still unknown.

Despite the difficulty in analyzing a large dataset of expression protocols, some general experimental rules have emerged. Our analysis revealed that IPTG concentration and growth temperature are important parameters that are complementary to the choice of a bacterial host. Plasmid usage analysis revealed that high copy number plasmids were preferentially used with C41λ(DE3), consistent with the fact that this mutant host was selected using a high copy number plasmid (pMW7^40^ encoding the oxoglutarate mitochondrial carrier^45^). Altogether, our data highlight that most of the strategies developed to improve expression systems have focused on limiting the toxicity for the bacterial host. Dong and co-workers were the first to show that in both *tac* and T7 based expression systems, and in presence of high copy number expression plasmids, gratuitous over-production of proteins leads to 16S ribosomal RNA destruction and loss of protein translation capacity, which is inversely correlated with the production level of the target protein. Restoring the fitness of the bacteria not only increases the yield of the over-produced protein but also impacts the folding and targeting of the over-produced protein. For instance, fine tuning of the target gene mRNA accumulation impacted the folding efficiency of the protein, which is exemplified by the production of AtpF in C43λ(DE3) membranes^19^. This initial findings with a simple bitopic *E. coli* membrane protein is now re-enforced by the success of C41λ(DE3) and C43λ(DE3) bacterial hosts’ ability to produce more complex α-helical MP. The choice of the appropriate bacterial host should thus rely on the viability of the cells^46^. In practice, a high copy number vector should be used in combination with the C41λ(DE3) host to take advantage of the strength of the T7 based expression system whereas, for more difficult MP, the C43λ(DE3) host, especially in combination with low copy number plasmids, offers the possibility to strongly attenuate the transcription of the target gene.

Our analysis of the PDB shows that they are very few mammalian MP produced in *E. coli* for structural studies. Two approaches have been developed to achieve eukaryotic MP production in *E. coli*. The first one involves engineering the bacterial host to improve its fitness during MP overproduction. Skretas and co-workers used GFP monitoring^47^ and cell sorting to identify genes (*nagD, nlpD, ptsN-rapZ-npr),* which, when co-expressed in multi-copy vector, enhances GPCR heterologous production in bacteria. They helped maintain periplasm and cell-envelope integrity, which in turn increased the folding efficiency of the newly synthetized GPCR^48^. The second approach is based on increasing the amount of correctly folded protein either by random or site directed mutagenesis. Sarkar *et al.* have elegantly addressed this challenge with the neurotensin receptor (NTR1)^49^. They have expressed a library of plasmids encoding random variants of the neurotensin receptor in *E. coli*. Using fluorescent ligands and cell sorting, they could identify mutants of NTR1 exhibiting a higher level of production, not only in *E. coli* but in yeast and mammalian cells as well. These mutants were also thermoresistant, which points to a common requirement for *in vivo* folding, membrane insertion and thermostability. Here we propose a third approach that could be developed together with the two strategies mentioned above. Pechman and Frydman^50^ proposed that codon optimality regulates the rhythm of translation elongation to ensure the efficiency of cotranslational folding of the peptide. In the case of MP, this principle could be applied not only to the cotranslational folding of the peptide but also to its interaction with the membrane targeting machinery^51^. The fact that eukaryotic MP do not follow the same translation rhythms as prokaryotic ones, could also explain why eukaryotic MP often aggregate as inclusion bodies when overproduced in *E coli*. Adapting codon optimality of eukaryotic MP genes to the *E. coli* translation dynamic could help prevent inclusion body formation, and this, together with the practical rules that have emerged from this study, could reveal the way in which the sequence space coverage of membrane proteome production in *E. coli* could be extended to eukaryotic sequences.

## Materials and Methods

### Analysis of membrane protein structure databases

Since NMR or crystal structure determination of proteins usually require milligram amounts of pure protein, this was used as a criterion to assess the success of expression systems. MP structures were extracted from the Protein Data Bank, by using the crystal structure database, interface and search engine developed by Steve White^2^ and the NMR structure database developed by Dror Warschawski^3^. Only accession codes referring to MP produced from *E. coli* were kept. Secondary accession numbers (structure obtained in presence of inhibitors or ligands, single mutations, changes in crystallisation conditions) were merged into one single entry. Note that when a MP structure was obtained using two different expression systems or bacterial hosts, both accession codes were kept. All related articles were downloaded and screened for expression hosts and vectors.

### Construction of the literature database

Articles citing Miroux and Walker^17^ for the use of the C41λ(DE3) and C43λ(DE3) hosts (group 1) Moffatt and Studier^13^ for the use BL21λ(DE3) host (group 2) and Guzman *et al.*^20^ for the use of the arabinose expression system (group 3) were downloaded from PubMed. The PMID list of each control group was converted to a PMCID (unique reference number for PubMed) list using the online PubMed PMCID/PMID/NIHMSID Converter. After conversion into text files of the downloaded articles, group 1 contained 756 articles out of 876 listed through the Web of Knowledge (WoK, using the INIST-CNRS access gate). Groups 2 and 3 consisted of 1005 and 1056 free access articles, respectively (Table 2). The text files for all three groups can be downloaded at https://www.dropbox.com/sh/cf6z7bj3k1sxegg/AADU1JLQ4fW0aeG_-HgtIqsCa?dl=0.

### Parsing of the literature database

The literature database was parsed for singular keywords or a combination of regular expressions. One positive hit per article was sufficient to select the articles of interest. Temperature and IPTG queries required manual annotation to avoid the counting of misleading hits.

### Temperature, inducer and plasmid search

In the global temperature search, all temperatures were recovered: growth temperature, as well as storage, centrifugation and denaturation temperatures, searching for a regular expression of the form of one, two or three digits preceded by a minus or a space and followed by a Celsius or a space then a c; transcribed into: ‘(\s|-)([0–9]*)(\s|°)c. Common and repetitive expressions were transcribed into a regular expression of the form *‘(harvested or cultured or grown or cultivated) at’* preceding the explicit temperature value. The specific temperature search targeted only temperatures of growth. Regarding IPTG induction, the line containing the term ‘iptg’ was recovered, and the explicit amounts of IPTG was recovered by a manual annotation. Distributions of IPTG concentrations and of temperatures of induction between groups were compared using ANOVA tests. P value <0.05 were considered as statistically significant. Plasmids were counted and classified in several groups depending on the origin of replication and on the presence of inhibiting sequences from the *lac* operon (*lacO*, *lacI* sequences) either within the expression plasmid or in companion plasmids. Plasmid nomenclature was not always consistent and plasmid names having lost their original nomenclature (pET, pRSET, pGEM, pMal etc…) could not be retrieved. Consequently, pET vectors are explicitly cited in only 40% and 33% of group 1 and 2, respectively. Plasmids encoding either lysozyme (pLysS/E), which has been shown to inhibit the T7 RNA polymerase, or encoding rare tRNA sequences, were also included in the search. T7 vectors were divided into three categories: pET based vectors, which are medium copy number plasmids (colE1 origin of replication); high copy number T7 based vectors (pMB1 origin of replication) and T7 attenuation vectors encoding either the *lacI* repressor or the lysozyme. Expression plasmids other than T7 based ones, such as pBAD, pcDNA and pRARE codon, were counted separately. The pcDNA eukaryotic expression plasmid served as a negative control for the regular expression search.

## Additional Information

**How to cite this article**: Hattab, G. *et al.* *Escherichia coli* as host for membrane protein structure determination: a global analysis. *Sci. Rep.* **5**, 12097; doi: 10.1038/srep12097 (2015).

## Supplementary Material

Supplementary Information

## Figures and Tables

**Table 1:** Bacterial expression hosts for non *E. coli* MP structure determination.

Bacterial host	Promoter				
	T7	*ara*	T5	*tet*	*trp/tac*
BL21λ(DE3)	40	1	1	2	2
C43λ(DE3)	18	10	1		–
C41λ(DE3)	16				
BL21λ(DE3) pLysS	12	2	1		
BL21λ(DE3) CodonPlus^1^	7			1	1
BL21 Star λ(DE3)	1	1			
BL21λ(DE3) Rosetta pLysS	1				
BL21λ(DE3) Tuner	1				1
BL21Rosetta	2				
BL21(AI)		1			
BL21-Gold	1				
*BL21*-*T1*^R^		4			
Lemo21	1				
Origami B			1		
B834	1			1	1
BLR					1
DH10B/ TOP10		3			
XL1-Blue		1	4		1
DH5a				1	3
SG13009			2		
MC4100		1			
SCM6		1			
MC1061		2			
JM83				2	
M15			1		
KRX					1
JM109					1
Not specified	1				2
Other		1^3^		1	
Total	102	28	11	8	14
Total (%)	63	17	7	5	8

^1^BL21-CodonPlus-λ(DE3)-RIL(4), BL21-CodonPlus λ(DE3)-RP(4), BL21-CodonPlus λ(DE3)-RIPL(1), BL21-CodonPlus λ(DE3)(1), BL21-CodonPlus λ(DE3)-RIL-X(1).

^2^An *E. coli* K strain that contains a chromosomal copy of the T7 RNA polymerase gene under rhamnose promoter (Promega).

^3^*Pseudomonas aeruginosa* used as expression system.

**Table 2:** Bacterial expression hosts for *E. coli* MP structures determination.

Bacterial host	Promoter						
	T7	Native	*ara*	*tac*	T5	*tet*	Other
BL21λ(DE3)	28	2		3			
C43λ(DE3)	10		4				
C41λ(DE3)	7						
BL21λ(DE3) pLysS	6						
BL21λ(DE3) pRIL	1						
BL21Starλ(DE3)	1						
BL21λ(DE3)Star pLysS	1						
BL21λ(DE3) Tuner	2						
BL21-Gold	3						
B834 (DE3)	3		1			1	
XL1-Blue		2		1	1		
*DH5-*α		2					1
JM109		1			1		
TOP10		1		1			
BZB1007		2					
HN705		1					
LS6164			1				
LE392			1				
UT5600							1
WH1061				1			
MEG119		1					
LMG194			1				
HN741				1			
LCB2048				1			
AW740		1					
RK20				1			
MH225		1					
TNE012		1					
FT004		1					
DW35		1					
MC4100		1					
GO105		1					
Not found							
Total	62	19	8	9	2	1	2
Total (%)	60	18	8	9	2	1	2

**Table 3:** Analysis of the bibliographic database.

**Group of articles**	**1 (T7)**	**2 (T7)**	**3 (*****ara)***
Number of articles, Web of Knowledge^1^	876	4626	2310
Unique articles converted and analysed	756	1056^2^	1005^2^
Citation of membrane protein (%^3^)	77	25	45
Articles with explicit amount of inducer	393	539	633
Article with explicit growth temperature values	256	493	526
Frequency of expression vector citation (%)			
pET3,^4^ vectors	40	34	19
pET vectors Δ(*lacI/lacO*)^3^,^5^	13	13	4
High copy number vectors Δ(*lacI/lacO*)^3^,^6^	19	13	8
T7 attenuation vectors3,^7^	10	16	4
pRARE codon vectors^3^	2	0.4	0.5
Vectors other than T7 based3,^8^	25	13	99^9^

^1^Number of citing articles at time of study (July 2012).

^2^Free access articles only.

^3^Frequency of word pattern count within the group of articles, limited to 1 match per article.

^4^pET (Novagen) are medium copy number plasmids (20–50/cell).

^5^pET(3, 9, 14, 17, 20, 23).

^6^pMW7 and derivatives (pHis and pRun), pGEM (Promega), pRSETand pDEST (Invitrogen), pIVEX (5prime), and pPR-IBA (IBA) are all high copy number plasmids (200–600/cell).

^7^p*lacI*, p*lysS*, p*lysE* (Novagen).

^8^pGEX (GE Healthcare), pASK (IBA), pQE (Quiagen), pMAL (New England Biolabs) and pBAD (Invitrogen-life technologies).

^9^pBAD exclusively.

**Table 4:** Distribution of plasmids in C41λ(DE3) and C43λ(DE3) hosts by most frequent users.

Laboratory	Number of articles in group 1^17^	Bacterial host vector combination			
		C41λ(DE3)			C43λ(DE3)
		pRSET	pHis/pMW7	pET	pET
Fersht A.R.	27	20		2	
Lowe J.	20	3	15	2	
Clarke J.	18	8	7		
Bycroft M.	17	13			
De la Cruz F.	12			6	3
Winkler H.H.	10			4	2
Dimroth P.	10				7
Suh S.W.	10				4
Total	124	44	22	14	16
Distribution (%)		35	18	11	13

^1^Origin or name of the vector not specified.

^2^pIVEX, pGEM, pMAL-C41λ(DE3).

^3^pUC8.

^4^pMal/C41λ(DE3).
