# Supplementary material for: Escherichia coli as host for membrane protein structure determination: a global analysis
Source: Sci Rep. 2015 Jul 10;5:12097. doi: 10.1038/srep12097 (PMC4498379; doi:10.1038/srep12097)
Supplement: Supplementary Information [file srep12097-s1.pdf]

Escherichia coli **as host for membrane protein structure determination: a global analysis.**

Georges Hattab, Dror E. Warschawski, Karine Moncoq and Bruno Miroux

Laboratoire de Biologie Physico-Chimique des Protéines Membranaires, Institut de Biologie Physico-Chimique, CNRS, Univ Paris Diderot, Sorbonne Paris Cité, PSL research university, Paris, France

Correspondance address :

Bruno.Miroux@ibpc.fr and Karine.Moncoq@ibpc.fr

Laboratoire de Biologie Physico-Chimique des Protéines Membranaires, UMR7099 CNRS IBPC, 13 rue Pierre et Marie Curie 75005 Paris, France

Telephone: 33 1 58 41 52 25

Fax: 33 1 58 41 52 24

Keywords:

structural biology, membrane protein, production of recombinant proteins, *Escherichia coli*, T7 RNA polymerase, bibliographic analysis.

Supplementary Figure-1 Miroux

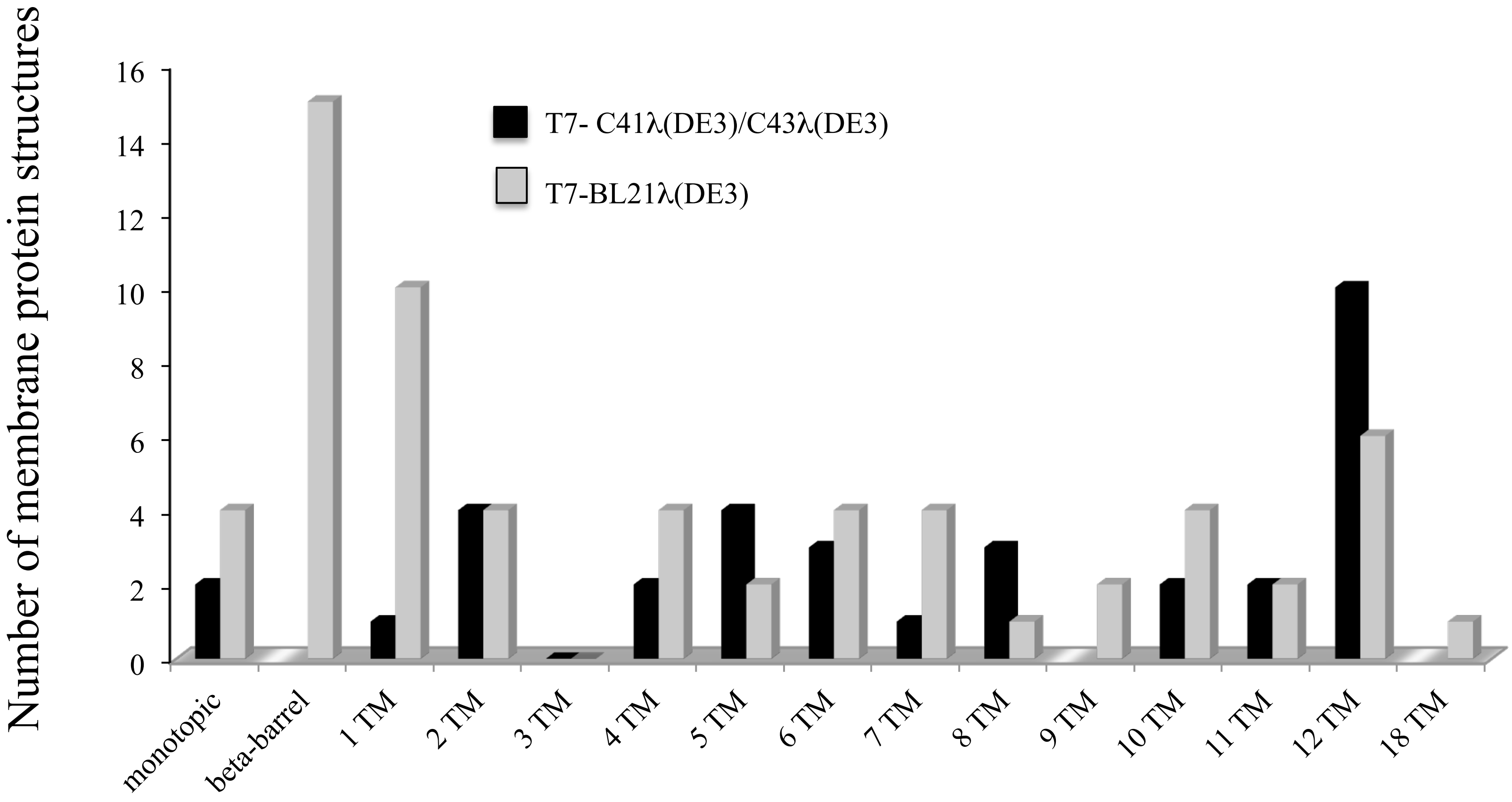

Supplementary Figure legend

Supplementary Fig. 1. Distribution of secondary structures in MP structures within the T7 expression system.

Membrane protein structures obtained from overexpression in the T7 system (102 see Table 1) were classified according to their secondary structure and topologies. For α-helical membrane proteins the number of transmembrane spans (TM) was represented from 1 to 18.

Supplementary Table 1-Miroux

List of non *E. coli* MP produced in *E. coli* host for structural studies.

**T7 expression system: C41(DE3) and C43(DE3) hosts**			
**PDB via Mpstruc (S. White)**	***Organism***	**Description**	**Name of bacterial host**
4EHW	*Aquifex aeolicus VF5*	Kinase (LpxK)	C41λ(DE3)
4GGM	*Bacillus thuringiensis serovar konkukian*	Phosphodiester hydrolase (LpxI)	C41λ(DE3)
2JO1	*Homo sapiens*	Phospholemman (FXYD1)	C43λ(DE3)
4F4L	*Magnetococcus marinus MC-1*	Bacterial voltage-gated sodium channel pore	C41λ(DE3)
2A9H	*Streptomyces lividans*	Channel (KcsA)	C41λ(DE3)
1WAZ	*Morganella morganii*	Transporter (MerF)	C43λ(DE3)
4LTO	*Alkalilimnicola ehrlichii*	Pore-only volt-gated sodium channel (NaVAe1p)	C41λ(DE3)
3P50, 3P4W, 3EAM, 3IGQ	*Gloeobacter violaceus*	Pentameric ligand-gated ion channel (GLIC)	C43λ(DE3)
3ZKR	*Erwinia chrysanthemi*	Prokaryotic pentameric ligand-gated ion channel (ELIC) in complex with bromoform:	C43λ(DE3)
4HZU	*Lactobacillus brevis*	ECF transporter complex	C43λ(DE3)
4A2N	*Methanosarcina acetivorans*	Transferase (ICMT)	C41λ(DE3)
3KP9	*Synechococcus sp.*	Thioredoxin domain protein (VKORC1)	C43λ(DE3)
2YVX, 2ZY9	*Thermus thermophilus HB8*	Transporter (MgtE)	C41λ(DE3)
3UX4	*Helicobacter pylori J99*	Channel (urea)	C43λ(DE3)
3S0X	*Methanococcus maripaludis*	Preflagellin aspartyl protease (FlaK)	C43λ(DE3)
3ODJ	*Haemophilus influenzae*	Peptidase (GlpG)	C43λ(DE3)
4JQ6	Isolated from the Mediterranean Sea	Proteorhodopsin (blue-light absorbing), Med12BPR.	C43λ(DE3)
3TIJ	*Vibrio cholerae*	Nupc family protein	C41λ(DE3)
4CAD	*Methanococcus maripaludis*	Protease Rce1 (CAAX)	C41λ(DE3)
3RFU	*Legionella pneumophila*	Copper efflux ATPase	C43λ(DE3)
4J72	*Aquifex aeolicus*	Phospho-MurNAc-pentapeptide translocase (MraY)	C41λ(DE3)
3ZUY	*Neisseria meningitidis*	Bacterial ASBT homologues	C43λ(DE3)
3WAJ	*Archaeoglobus fulgidus*	OST with bound zinc & sulfate (AglB)	C43λ(DE3)
2B2F	*Archaeoglobus fulgidus*	Transporter (Amt-1)	C43λ(DE3)
3F3A, 2QEI, 2A65, 3GJD, 3MPN, 3TT1, 3USG 3QS4, 4MM4	*Aquifex aeolicus VF5*	Symporter (LeuT)	C41λ(DE3)
4IKV	*Geobacillus kaustophilus*	Proton-dependent oligopeptide transporter (POT)	C41λ(DE3)ΔacrB
4LDS	*Staphylococcus epidermidis*	Glucose/H+ symporter (GlcP)	C41λ(DE3)
3VVN	*Pyrococcus furiosus*	Multidrug and Toxin Compound Extrusion (MATE)	C41λ(DE3)ΔacrB
4KPP	*Archaeoglobus fulgidus*	Ca2+/H+ antiporter (CaX)	C41λ(DE3)
4K0J	*Cupriavidus metallidurans*	ZneA Zn(II)/proton antiporter	C43λ(DE3)
3GIA	*Methanocaldococcus jannaschii*	protein MJ0609 (ApcT)	C41λ(DE3)
4APS	*Streptococcus thermophilus*	Transporter (PepTSo)	C43λ(DE3)
2XUT	*Shewanella oneidensis*	Transporter (PepTSt)	C43λ(DE3)
2V50	*Pseudomonas aeruginosa*	Transporter (MexB)	C43λ(DE3)

**T7 expression system: BL21(DE3) derivatives**			
**PDB via Mpstruc (S. White)**	***Organism***	**Type**	**Strain(s)**
3L7I	*Staphylococcus epidermidis*	Polymerase (TagF)	BL21λ(DE3)
4G9K	*Saccharomyces cerevisiae*	NADH dehydrogenase (Ndi1)	BL21λ(DE3)pLysS
3VV9	*Trypanosoma brucei brucei*	Alternative oxidase (AOX)	BL21λ(DE3)
2XCI	*Aquifex aeolicus*	Glycosyltransferase (WaaA, KdsB)	BL21CodonPlusλ(DE3)
3RBH	*Pseudomonas aeruginosa*	Export Protein (AlgE)	BL21CodonPlusλ(DE3)
1H6S, 1BH3	*Rhodopseudomonas blastica*	Porin (E1M/A116K)	BL21λ(DE3)pLysS
3FID	*Salmonella typhimurium*	lipid A deacylase (LpxR)	BL21Starλ(DE3)
4E1T	*Yersinia pseudotuberculosis*	Invasin beta-domain	BL21λ(DE3)
3V8X	*Neisseria meningitidis*	Transferrin (TbpA)	BL21λ(DE3)
3QRA	*Yersinia pestis*	Adhesion Protein (Ail)	BL21λ(DE3)
3A2R	*Neisseria meningitidis*	OuterMP (PorB)	BL21λ(DE3)
2K0L (lsNMR)	*Klebsiella pneumoniae*	OuterMP (OmpA)	BL21λ(DE3)
2K4T (lsNMR)	*Homo sapiens*	Anion channel (VDAC-1)	BL21λ(DE3)
3EMN	*Mus musculus*	Anion channel (VDAC-1)	BL21λ(DE3)
2QDZ	*Bordetella pertussis*	Transporter (FhaC)	BL21λ(DE3)
1UYN	*Haemophilus influenzae*	Autotransporter (Trimeric Hia)	BL21λ(DE3)
3B07	*Staphylococcus aureus*	γ-hemolysin composed of LukF and Hlg2	B834λ(DE3)
4K3B	*Neisseria gonorrhoeae*	BamA with POTRA domains 1 - 5	BL21λ(DE3)
4K3C	*Haemophilus ducreyi*	BamA with POTRA domains 4 & 5	BL21λ(DE3)

2LOU	*Homo sapiens*	Apelin receptor	BL21λ(DE3)
2MFR	*Homo sapiens*	Insulin receptor TM domain (AAs 940-980)	BL21λ(DE3)
3VMT	*Staphylococcus aureus*	Glycosyltransferase	BL21CodonPlusλ(DE3)
2KS1, 2JWA (lsNMR)	*Homo sapiens*	ErbB1/ErbB2, TM heterodimer	BL21λ(DE3)pLysS
2LZL (lsNMR)	*Homo sapiens*	Fibroblast growth factor receptor 3 (FGFR3, TM)	BL21λ(DE3)pLysS
2LCX (lsNMR)	*Homo sapiens*	ErbB4, TM dimer	BL21λ(DE3)pLysS
2L0J, 2LY0, 2KIX	*Influenza A*	Channel (AM2)	BL21CodonPlusλ(DE3) and BL21λ(DE3)pLysS
2KYV	*Homo sapiens*	Phospholamban Homopentamer	BL21λ(DE3)
2HAC (lsNMR)	*Homo sapiens*	TCR-CD3, TM dimer complex	BL21λ(DE3)
2M8R , 3HD7	*Rattus norvegicus*	Syntaxin 1A, TM & Syntaxin complex	BL21λ(DE3)
2HN2	*Thermotoga maritima*	Transporter (CorA Mg2+)	BL21CodonPlusλ(DE3)
2H3O	*Morganella morganii*	Transporter (MerF HgII)	BL21λ(DE3)pLysS
4EV6	*Methanocaldococcus jannaschii*	Transporter (CorA Mg2+)	BL21λ(DE3)Rosetta
3ZRS, 2WLJ, 4LP8	*Magnetospirillum magnetotacticum*	Channel (KirBac3.1)	BL21CodonPlusλ(DE3)
3DWW (electron microscopy)	*Homo sapiens*	human microsomal prostaglandin E synthase 1	BL21λ(DE3)pLysS
2XQA, 3EHZ, 4HFI	*Gloebacter violaceus*	Channel (pentameric GLIC)	BL21λ(DE3)
2VL0	*Herpes simplex*	Channel (pentameric ELIC)	BL21λ(DE3)
2Q7M	*Homo sapiens*	Lipoxygenase protein (FLAP)	BL21λ(DE3)
2M6B	*Homo sapiens*	Human glycine receptor (hGlyR-α1), TM monomer)	BL21λ(DE3)pLysS
4HTS	*Aquifex aeolicus*	Twin-arginine translocase receptor (TatC)	BL21λ(DE3)
4HUQ	*Lactobacillus brevis*	Folate ECF transporter complex	BL21λ(DE3)
3K3F	*Desulfovibrio vulgaris*	Transporter (Urea)	BL21λ(DE3)
3B60	*Salmonella typhimurium*	Flippase (MsbA)	BL21λ(DE3)
4DXW	*Alpha proteobacterium himb114*	Na channel (NaV)	BL21λ(DE3)
3P5N	*Staphylococcus aureus*	Transporter (RibU)	BL21λ(DE3)
4J9U	*Vibrio parahaemolyticus*	Potassium ion transporter (TrkH) in complex with TrkA	BL21λ(DE3)
2LNL (ssNMR)	*Homo sapiens*	Receptor (CXCR1)	BL21λ(DE3)
3QDC, 2KSY, 1H68, 1H2S	*Natronomonas pharaonis*	Rhodopsin (SRII)	BL21λ(DE3) and BL21λ(DE3) Tuner
4HYJ	*Exiguobacterium sibiricum*	Proteorhodopsin: Lysine is the proton donor in this novel proteorhodopsin	Rosettaλ(DE3)pLysS
4J7C	*Bacillus subtilis*	Potassium ion transporter (KtrAB)	BL21λ(DE3)
4HYG	*Methanoculleus marisnigri*	Presenilin(PSH)	BL21λ(DE3)
4HG6	*Rhodobacter sphaeroides*	Cellulose synthase/cellulose translocation intermediate (BcsA-BcsB)	Rosetta 2
3QNQ	*Bacillus cereus*	Transporter (ChbC EIIC)	BL21λ(DE3)
3PJZ	*Vibrio parahemolyticus*	Transporter (TrkH)	BL21λ(DE3)
4G1U	*Yersinia pestis*	Transporter (HmuUV)	BL21Goldλ(DE3)
3M71	*Haemophilus influenzae*	Anion channel (SLAC1)	BL21λ(DE3)pLysS
4KJS	*Bacillus subtilis*	Ca2+/H+ antiporter (YfkE)	BL21λ(DE3)
4F35	*Vibrio cholerae*	Symporter	BL21λ(DE3)
3AQP	*Thermus thermophilus*	(SecDF)	BL21CodonPlusλ(DE3)
4LZ6	*Bacillus halodurans*	Multidrug and Toxin Compound Extrusion (MATE) transporter (DinF-BH)	BL21λ(DE3) ΔacrABΔmacABΔyojHI
4BWZ	*Thermus thermophilus*	Na+/H+ antiporter (NapA)	Lemo21λ(DE3)
4M8J	*Proteus mirabilis*	Carnitine transporter (CaiT)	BL21λ(DE3)pLysS
4HUK	*Neisseria gonorrhoeae*	Multidrug and Toxin Compound Extrusion (MATE) transporter (NorM)	BL21λ(DE3)
3MKT	*Vibrio cholerae*	Transporter (NorM & MATE)	BL21λ(DE3)
1KPL	*Salmonella typhimurium*	Transporter (H+/Cl-)	BL21λ(DE3)
**Arabinose promoter based expression system**			
**PDB via Mpstruc (S. White)**	***Organism***	**Type**	**Strain(s)**
3SY7, 2SY9, 3SYB	*Pseudomonas aeruginosa*	Channel (OccD1, OprD)	BL21λ(DE3)T1phage resistant
3SZD	*Pseudomonas aeruginosa*	Channel (OccK2, OpdF)	BL21λ(DE3)T1phage resistant
3SZV, 3T0S, 3T20, 3T24	*Pseudomonas aeruginosa*	Aromatic Hydrocarbon (OccK3, OpdO)	BL21λ(DE3)T1phage resistant
4GEY	*Pseudomonas putida*	Carbohydrate-specific transporter (OprB)	BL21λ(DE3)T1phage
2LHF	*Pseudomonas aeruginosa*	Outer MP (OprH)	PA(Δ*oprH)*
2X27	*Pseudomonas aeruginosa*	Outer MP (OprG)	C43λ(DE3)
2X55	*Yersinia pestis*	Plasminogen activator (Pla)	C43λ(DE3)
3D5K	*Pseudomonas aeruginosa*	Outer MP (OprM)	C43λ(DE3)
2QTK	*Pseudomonas aeruginosa*	Benzoate channel (OpdK)	C43λ(DE3)
2ODJ	*Pseudomonas aeruginosa*	Channel (OprD)	C43λ(DE3)

3KVN	*Pseudomonas aeruginosa*	Autotransporter (EstA)	C43λ(DE3) & BL21Starλ(DE3)
3CSL	*Serratia marcescens*	Heme receptor complex (HasR)	MC4100 derivative
3DWO	*Pseudomonas aeruginosa*	Fatty acid transporter (FadL)	C43λ(DE3)
3TDO	*Clostridium difficile*	Hydrosulfide Ion Channel (FNT3)	BL21λ(DE3)pLysS
3ODJ, 2NR9	*Haemophilus influenzae*	IntraMembrane peptidase (GlpG)	TOP10
3KLY	*Vibrio cholerae*	Formate transporter (FocA)	C43λ(DE3)
3QF4	*Thermotoga maritima*	Heterodimeric ABC exporter	C43λ(DE3) or MC1061
1XFH, 3KBC, 3V8F	*Pyrococcus horikoshii*	Glutamate Transporter Homologue (GltPh)	TOP10 or DH10B
4KY0	*Thermococcus kodakarensis*	Aspartate Transporter	MC1061
3DL8	*Aquifex aeolicus*	Channel (SecYEG)	C43λ(DE3)
3MP7	*Pyrococcus furiosus*	Primed channel (SecYEb)	BL21λ(DE3)AI
3DIN	*Thermotoga maritima* MSB8	SecYEG protein in complex SecA	BL21λ(DE3)
2QJU	*Aquifex aeolicus*	Leucine Transporter (LeuT)	BL21λ(DE3)pLysS
3RCE	*Campylobacter lari*	OST in complex (PglB)	BL21-Gold SCM6
3DH4	*Vibrio parahaemolyticus*	NaGalactose Transporte (ySGLT)	XL1-blue
2XQ2	*Vibrio parahaemolyticus*	NaGalactose Transporter (ySGLT)	TOP10
**T5 promoter based expression system**			
**PDB via Mpstruc (S. White)**	***Organism***	**Type**	**Strain(s)**
4HHS	*Arabidopsis thaliana*	Fatty acid α-dioxygenase (α-DOX)	M15
3O44	*Vibrio cholerae*	Pore-forming toxin (Cytosolin)	Origami B
4G6G	*Saccharomyces cerevisiae*	Dehydrogenase (Ndi1)	C43λ(DE3)
4GX0	*Geobacter sulfurreducens*	Channel (GsuK)	BL21λ(DE3)
3RBZ, 4EI2 4HYO, 4L73, 1LNQ, 3LDC,	*Methanothermobacter thermautotrophicus*	Potassium channel and mutants (MthK)	XL1-Blue, SG1309
4H33	*Listeria monocytogenes*	Channel (KyLm)	XL1-Blue
3STL, 3OR7, 3EFF, 1BL8	*Streptomyces lividans*	Channel (KcsA)	XL1-Blue
3E86, 3KOD, 3OUF, 3T1C, 3E86, 2Q67, 2AHY	*Bacillus cereus*	Channel (NaK)	SG13009
1ORQ	*Aeropyrum pernix*	Channel (KyAP)	XL1-Blue
3V5U	*Methanocaldococcus janaschii*	Exchanger (NaCa)	BL21λ(DE3)pLysS
**Tetracyclin promoter based expression system**			
**PDB via Mpstruc (S. White)**	***Organism***	**Type**	**Strain(s)**
2LME	*Yersinia enterocolitica*	Autotransporter (trimeric YadA)	BL21λ(DE3)
2GR8	*Haemophilus influenzae*	Trimeric autotransporter (Hia)	B834
3IGA	*Streptomyces lividans*	Potassium channel (KcsA)	JM-83
3BEH	*Mesorhizobium lotiMloti*	K1 cyclic nucleotide-regulated K+-channel	JM83
4DOJ, 2WIT	*Corynebacterium glutamicum*	Glycine betaine transporter (BetP)	DH5a<br>BL21 RIL-Xλ(DE3)
3NCY	*Salmonella enterica*	Antiporter (AdiC)	BL21λ(DE3)
3NDO	*Synechocystis sp.* pcc 6803	H+/Cl- Eukaryotic Exchange Transporter	variant strain from Stratagene lot # 0420399
**Other promoter (Tac, Trp, Rham) based expression system**			
**PDB via Mpstruc (S. White)**	***Organism***	**Type**	**Strain(s)**
1LKF	*Staphylococcus aureus*	Component of γ-hemolysin (LukF)	B834 and DH5a
4HSC	*Streptococcus pyogenes*	Streptolysin O pore-forming toxin	XL-1 Blue
1CWV	*Yersinia pseudotuberculosis*	Invasin C-terminal passenger domain	not found
2M6X (lsNMR)	*Hepatitis C virus*	p7 hexamer channels (isolate EUH1480)	DH5α
1ZLL, 2M3B	*Homo sapiens*	Phospholamban homopentamer	BL21λ(DE3)
2KNC (lsNMR)	*Homo sapiens*	Human Integrin αIIbβ3 transmembrane-cytoplasmic heterodimer	BL21λ(DE3)
3VOU	*Bacillus weihenstephanensis (NaK) and Sulfitobacter pontiacus (NaV)*	NaK channel chimera with grafted C-terminal region of a NaV channel	KRX strain (Promega)
2F2B	*Methanothermobacter marburgensis*	Aquaporin water channel (AqpM)	not found
4BUO	*Rattus norvegicus*	Neurotensin receptor produced by direct evolution (NTS1)	BL21 TUNER
2M3G (ls NMR)	*Anabaena (Nostoc)* sp. PCC7120	Sensory Rhodopsin	BL21-Codonplus-RIL
2JLN	*Microbacterium liquefaciens*	Benzyl-hydantoin transporter (Mhp1)	BLR (Novagen)
4C7R	*Corynebacterium glutamicum*	Glycine betaine transporter (BetP)	DH5α
3W9J	*Pseudomonas aeruginosa*	Bacterial multi-drug efflux transporter (MexB)	JM109

Supplementary Table 2-Miroux

List of *E. coli* MP produced in *E. coli* hosts for structural studies

PDB via Mpstruc (S. White)	Type of membrane proteins	Name of bacterial host
	MONOTOPIC	
1B12	Signal Peptidase (SPase) in complex with a β-lactam inhibitor	BL21λ(DE3)
2QCU	Glycerol-3-phosphate dehydrogenase (GlpD, native).	XL1blue/JM109
1J79, 1XGE	Dihydroorotate Dehydrogenase.	XL1blue
	BETA BARRELS	
1MPF	OmpF Porin from colicin-resistant.	BZB1107
1GFM	OmpF Porin, D113G mutant.	Top10
1BT9, 3O0E	OmpF Porin, D74A mutant.	BL21λ(DE3)
1HXX	OmpF Porin, Y106F Mutant.	BL21λ(DE3)Domp8
3HW9, 2ZFG	OmpF Porin	MH225
2J1N	OmpC Osmoporin.	BZB1107
2XE1	OmpC Osmoporin clinical variant OmpC06.	HN705 Domp8
2F1C	OmpG *monomeric* porin.	C43λ(DE3)
2IWW	OmpG *monomeric* porin.	C41λ(DE3)
2JQY	OmpG by solution NMR spectroscopy.	BL21λ(DE3)pLysS
1MPM, 1MAL, 1AF6	LamB Maltoporin in complex with maltose.	K12 pop65lO
1EK9, 1TQQ	Outer membrane protein TolC central to multidrug efflux and protein export.	BL21λ(DE3)
2VDE	TolC outer membrane protein (Y362F, R367E), partially open state.	C43λ(DE3) and C41λ(DE3)
3PIK, 4K7R, 4K7K, 4K34	CusC, the Outer Membrane Component of a Heavy Metal Efflux Pump.	C43λ(DE3)/BL21Starλ(DE3)
1NQE/F/G/H	BtuB cobalamin transporter.	not found
1UJW	BtuB with bound colicin E3 R-domain.	TNE012 (K12 tsx−ompA− ompB−)
2GSK	BtuB:TonB.	BL21Starλ(DE3)pLysS
2YSU	Complex of the Colicin E2 R-domain and Its BtuB Receptor.	TNE012 (K12 tsx−ompA− ompB−)
2HDI	Colicin I receptor Cir in complex with Colicin Ia binding domain.	BL21λ(DE3)
1QJP/1BXW, 1G90	OmpA.	BL21λ(DE3)
2JMM	OmpA with four shortened loops: NMR Structure DHPC micelles.	BL21λ(DE3)Gold
1I78	OmpT outer membrane protease.	BL21λ(DE3), DH5α and B884
2F1V	OmpW outer membrane protein.	C43λ(DE3)
1ORM, 1Q9F	OmpX: , NMR (DHPC micelles).	BL21λ(DE3)pLysS
2MO6	OmpX in optimized nanodiscs: NMR Structure In DPC micelles.	BL21λ(DE3)
1FW2	OmpLA (PldA) outer membrane phospholipase A monomer with Ca++.	BL21λ(DE3)
1ILZ	OmpLA (PldA) active-site mutant (N156A).	BL21λ(DE3)ΔpldA
2WJR	NanC Porin, model for KdgM porin family.	BL21λ(DE3)pLysS
1MM4, 1MM5, 1THQ, 3GP6	PagP outer membrane palimitoyl transferease.	BL21λ(DE3)
1T16, 3PGR	FadL long-chain fatty acid transporter.	C43λ(DE3)
3DWN	FadL long-chain fatty acid transporter A77E/S100R mutant.	Ls6164, C43λ(DE3)
1BY3	FhuA, Ferrichrome-iron receptor without ligand. With ligand: 1BY5.	B834(DE3)/BL21λ(DE3)
1QKC, 1FI1, 2FCP	FhuA in complex with albomycin.	AW740 [DompF zch:TnlO DotnpCfltuA31]
1FCP	SeMet-FhuA.	DL41
2GRX	FhuA in complex with TonB.	AW740
1FEP, 1PNZ	FepA, Ferric enterobactin receptor.	BL21λ(DE3)
1KMO	FecA, siderophore transporter.	UT5600
2VQI	P pilus usher translocation domain.	B834λ(DE3)
3RFZ	P pilus FimD usher bound to FimC:FimH substrate.	B834λ(DE3)
4J3O	P pilus FimD usher in complex with FimC:FimF:FimG:FimH.	Tunerλ(DE3)
2YNK	Wzi outer-membrane lectin.	Top10/B834λ(DE3)
2QOM, 3SLJ, 3SZE	EspP autotransporter.	BL21λ(DE3)
3AEH	Hbp (hemoglobin protease) self-cleaving autotransporter with truncated passenger.	C43λ(DE3)
1WXR	Hbp (hemoglobin protease) full-length passenger domain.	DH5α
4-E1S, 1F02	Intimin outer membrane β-domain.	BL21λ(DE3)
4C00	TamA Autotransporter, full length.	BL21λ(DE3)
	TRANSMEMBRANE PROTEINS: ALPHA-HELICAL	
3FWM	Peptidoglycan Glycosyltransferase penicillin-binding protein 1b.	BL21λ(DE3)
2WCD	Cytolysin A (ClyA, aka HlyE).	Tunerλ(DE3)
2J58	Wza translocon for capsular polysaccharides.	LE392
3JQO	Type IV outer membrane secretion complex.	B834λ(DE3)
2OAU, 2VV5	MscS voltage-modulated mechanosensitive channel.	BL21λ(DE3)
1RC2, 2O9D, 3NK5	AqpZ aquaporin water channel.	C43λ(DE3)
2ABM	AqpZ aquaporin showing two conformations of Arg-189.	BL21λ(DE3)pLysS
1FX8 , 1LDF	GlpF glycerol facilitator channel.	not found
3KCU	FocA, pentameric aquaporin-like formate transporter.	BL21λ(DE3)
1U7G	AmtB ammonia channel (mutant).	C41λ(DE3)
1XQF, 2NMR, 2NUU, 2NS1	AmtB ammonia channel.	C43λ(DE3)

3TXT, 3UBB, 2LEP, 4HDD	GlpG rhomboid-family intramembrane protease.	BL21λ(DE3)
2LZS	TatA, Twin arginine translocase.	BL21λ(DE3)pLysS
1OTS, 2EXW, 4FG6	H+/Cl- Exchange Transporter: Formerly ClC Chloride Channel.	BL21λ(DE3)
3NMO	Monomeric H+/Cl- Exchange Transporter.	stratagene
4ENE	H+/Cl- Exchange Transporter (truncated). Truncation: Residues 2-16 at N-terminal and 461-464 at C-terminal.	stratagene
1OY6	AcrB bacterial multi-drug efflux transporter.	DH5α
1T9T, 2GIF	AcrB bacterial multi-drug efflux transporter.	C43λ(DE3)
2HQC	AcrB bacterial multi-drug efflux transporter.	BL21λ(DE3)Gold
2DHH, 2RDD, 2W1B, 3AOB, 3W9H, 1IWG	AcrB bacterial multi-drug efflux transporter.	JM109
3K07, 3NE5	CusA metal-ion efflux pump.	BL21λ(DE3)
3OOC	CusB membrane fusion protein (apo protein).	DH5α
3B5D	EmrE bacterial multi-drug efflux transporter with bound TPP substrate.	BL21λ(DE3)
1PV7, 2CFQ	LacY Lactose Permease Transporter.	not found
2V8N	LacY Lactose Permease (wild-type) with TDG.	XL1blue
2Y5Y	LacY Lactose Permease with covalently bound MTS-gal.	C43λ(DE3)
4OAA	LacY Lactose Permease Transporter (G46W/G262W mutant) with bound lactose analog.	C41λ(DE3)
3O7Q	FucP Fucose Transporter in outward-facing conformation.	BL21λ(DE3)
1PW4	GlpT Glycerol-3-Phosphate Transporter.	LMG194
2GFP	EmrD Multidrug Transporter.	not found
4GBY	XylE proton:xylose symporter with bound D-xylose.	BL21λ(DE3)
4AJ3	XylE proton:xylose symporter in partially occluded inward-open state.	C41λ(DE3)
4IU9	NarU nitrate transporter.	not found
4JR9	NarK nitrate/nitrite exchanger. A member of the nitrate/nitrite porter family (NNP).	C41λ(DE3)
3WDO	YajR drug efflux transporter.	C43λ(DE3)
3QE7	Nucleobase/ascorbate transporter (NAT) UraA uracil/H+ symporter.	Bl21λ(DE3)
3HFX	CaiT carnitine transporter.	C41λ(DE3)
2WSX	CaiT carnitine transporter Fully-open inward-facing conformation.	BL21λ(DE3)
3LRB, 3L1L, 3OB6	AdiC Arginine:Agmatine Antiporter.	BL21λ(DE3)
4DJK	GadC glutamate-GABA antiporter.	not found
2QFI, 3H90	YiiP Zinc Transporter.	BL21λ(DE3)
1ZCD	NhaA Na+/H+ antiporte.	rk20
3FI1	NhaA Na+/H+ antiporter Difference maps show structural changes with changes in pH.	BL21λ(DE3)
1L7V, 2QI9	BtuCD Vitamin B12 Transporter.	BL21λ(DE3)
4FI3	BtuCD-F Vitamin B12 Transporter with bound AMP-PNP.	BL21λ(DE3)CodonPlusRIPL
2R6G, 4JBW, 3PV0	MalFGK2-MBP Maltose uptake transporter complex.	HN741
3RLF	MalFGK2-MBP Maltose uptake transporter complex with bound MgAMPPNP.	HN597 (DuncB-C i1ur:TnlO araD lac rpsLI/F' laca lacZ::Tn5, proA+ proB+)
4KHZ, 4KI0	MalFGK$_2$-MBP Maltose uptake transporter complex; pre-translocation conformation bound to maltoheptaose.	AD126, Top10F', BL21l(DE3)
3DHW	MetNI Methionine uptake transporter complex MetN-C2 domain.	BL21λ(DE3)Gold
3OAA	F1-ATPase in an autoinhibited conformation.	not found
1A91	Subunit C of the F1Fo ATP synthase.	MEG119
2KDC	Diacylglycerol kinase (DAGK). Domain-swapped homotrimer.	BL21λ(DE3) WH1061
3ZE4	Diacylglycerol kinase (DAGK).	not found
2QCU	Glycerol-3-phosphate dehydrogenase (GlpD, native).	XL1Blue
1Q16, 1SIW, 1Y4Z	NarGHI Nitrate Reductase A.	LCB2048
2LTQ	DsbB in POPE lipid bilayer: Cys41Ser mutant. Solid-state NMR used to refine the X-ray structure 2ZUQ.	C43λ(DE3)
4GD3, 3USE	O2-tolerant Hydrogenase-1 in complex with cytochrome b: Structure includes transmembrane helices.	FT004
3M9C, 3RKO	Electron Transport Chain Complexes I.	BL21λ(DE3)
1FUM	Electron Transport Chain Complexes: Complex II Fumarate Reductase Complex	not found
1L0V	Native Fumarate Reductase Complex: +HQNO.	DW35
1KQF	Formate dehydrogenase-N: HQNO complex.	GL101
1NEK, 2ACZ, 2WDQ, 2WP9	Succinate:quinone oxidoreductase (SQR, Complex II).	MC4100
1FFT	Electron Transport Chain Complexes: Complex IV (Cytochrome C Oxidase).	GO105 lacking terminal oxydases
